# ^1^H-MRS for characterizing metabolic alterations in HIV-positive patients with and without memory deficits

**DOI:** 10.1590/0100-3984.2020.53.6e1

**Published:** 2020

**Authors:** Fabiano Reis

**Affiliations:** 1 Associate Professor of Neuroradiology and Chair of the Neuroradiology Department at the Universidade Estadual de Campinas (Unicamp), Campinas, SP, Brazil. Email: fabianoreis2@gmail.com.

Proton magnetic resonance spectroscopy (^1^H-MRS) provides a noninvasive method for examining a wide variety of metabolites in the human brain^(1,2)^, including N-acetylaspartate (NAA), a compound present in neurons and axons; choline-containing compounds, which are involved in membrane synthesis and degradation; phosphocreatine and creatine, which play a major role in energy metabolism; lactate, a resultant of increased anaerobic glycolysis; lipids, which correlate with necrosis; and others, such as succinate, amino acids, glycine, glutamine, and myo-inositol. The acquisition of the localized metabolic information available from proton spectra can be correlated with magnetic resonance imaging (MRI) and other imaging methods, potentially leading to the development of a set of physiological, anatomical, and biochemical parameters, which may provide a powerful approach to investigating the underlying pathophysiology of many disorders^(1)^. Even in a brain with a normal appearance (in structural MRI sequences), such abnormalities can be observed. Technically, detecting the absolute concentrations from spectroscopy is relatively difficult. Therefore, metabolite-to-creatine ratios are calculated, which requires us to make an assumption that creatine concentrations remain constant and stable^(3)^. First, it is convenient and easy to acquire a ^1^H-MRS spectrum from a standard MRI scanner with a short echo time. The spectrum can be obtained either from a single voxel of interest or from multiple areas (multivoxel spectroscopy). That makes ^1^H-MRS simultaneously sensitive to metabolite changes in multiple regions^(4)^. In addition, ^1^H-MRS is a noninvasive, radiation-free technique that has the advantage of monitoring disease progression^(3)^.

The current issue of Radiologia Brasileira features an excellent article by Correa et al.^(5)^, entitled “Posterior cingulate gyri metabolic alterations in HIV-positive patients with and without memory deficits”, in which 36 HIV-positive patients (with and without memory deficits) were compared with 22 controls. Despite the small number of patients evaluated in their study, the authors demonstrated that choline/creatine ratios in the posterior cingulate gyri were significantly higher in the HIV-positive patients (with and without memory deficits) than in the controls. The authors also corroborated data presented in previous studies^(6-8)^, as well as underscoring the fact that there are direct and indirect mechanisms, as well as certain events, associated with HIV encephalitis. An elevated choline level may provide a useful marker for the early effects of HIV infection on the central nervous system before the onset of dementia and a reduction in NAA^(6)^.

Glycerophosphocholine (GPC) and phosphocholine (PC) represent the major constituents of the *in vivo* ^1^H-MRS-detected choline resonance, to which free choline makes only a minor contribution. If membrane damage occurs subsequent to HIV infection, choline-containing compounds (GPC, PC, and free choline) may be released, thus increasing the intensity of the choline signal^(9)^. Supporting this hypothesis is the finding of significant damage to and vacuolization of the dendritic tree in HIV encephalitis^(7)^. That may also reflect an increase in the number of macrophages and microglia in the brains of patients with AIDS. The use of ^1^H-MRS provides a sensitive, noninvasive means of detecting alterations in the microenvironment of HIV. A reduction in NAA occurs only in patients suffering from more advanced dementia, and an increased choline level may therefore be a more reliable marker of early brain abnormalities associated with HIV infection. In a previous study^(8)^, an elevated choline peak before the onset of dementia was demonstrated in HIV-infected patients and, as in the Correa et al. study^(5)^, was not found to correlate with neuropsychological performance.
